# Preventing swarm detection in extracellular vesicle flow cytometry: a clinically applicable procedure

**DOI:** 10.1016/j.rpth.2023.100171

**Published:** 2023-05-02

**Authors:** Naomi C. Buntsma, Mona Shahsavari, Aleksandra Gąsecka, Rienk Nieuwland, Ton G. van Leeuwen, Edwin van der Pol

**Affiliations:** 1 Amsterdam Vesicle Center and Laboratory of Experimental Clinical Chemistry, Amsterdam UMC, University of Amsterdam, Amsterdam, the Netherlands; 2 Biomedical Engineering & Physics, Amsterdam UMC, University of Amsterdam, Amsterdam, the Netherlands; 3 Department of Neurology, Amsterdam UMC, University of Amsterdam, Amsterdam, the Netherlands; 4 Amsterdam Cardiovascular Sciences, Atherosclerosis and Ischemic Syndromes, Amsterdam, the Netherlands; 5 Amsterdam Neuroscience, Neurovascular Disorders, Amsterdam, the Netherlands; 6 Cancer Center Amsterdam, Imaging and Biomarkers, Amsterdam, the Netherlands; 7 1st Chair and Department of Cardiology, Medical University of Warsaw, Warsaw, Poland; 8 Amsterdam Cardiovascular Sciences, Microcirculation, Amsterdam, the Netherlands

**Keywords:** biomarkers, exosomes, extracellular vesicles, flow cytometry, plasma

## Abstract

**Background:**

Flow cytometry is commonly used to detect cell-derived extracellular vesicles in body fluids such as blood plasma. However, continuous and simultaneous illumination of multiple particles at or below the detection limit may result in the detection of a single event. This phenomenon is called swarm detection and leads to incorrect particle concentration measurements. To prevent swarm detection, sample dilution is recommended. Since the concentration of particles differs between plasma samples, finding the optimal sample dilution requires dilution series of all samples, which is unfeasible in clinical routine.

**Objectives:**

Here we developed a practical procedure to find the optimal sample dilution of plasma for extracellular vesicle flow cytometry measurements in clinical research studies.

**Methods:**

Dilution series of 5 plasma samples were measured with flow cytometry (Apogee A60-Micro), triggered on side scatter. The total particle concentration between these plasma samples ranged from 2.5 × 10^9^ to 2.1 × 10^11^ mL^−1^.

**Results:**

Swarm detection was absent in plasma samples when diluted ≥1.1 × 10^3^-fold or at particle count rates <3.0 × 10^3^ events·s^-1^. Application of either one of these criteria, however, resulted in insignificant particle counts in most samples. The best approach to prevent swarm detection while maintaining significant particle counts was by combining minimal dilution with maximum count rate.

**Conclusion:**

To prevent swarm detection in a series of clinical samples, the measurement count rate of a single diluted plasma sample can be used to determine the optimal dilution factor. For our samples, flow cytometer, and settings, the optimal dilution factor is ≥1.1 × 10^2^-fold, while the count rate is <1.1 × 10^4^ events·s^−1^.

## Essentials

• Swarm detection affects concentration measurements in extracellular vesicle flow cytometry.
• Sample dilution can prevent swarm detection but should not result in a loss of particle counts.
• We found that swarm detection can be prevented by use of a minimum dilution and a maximum count rate.
• The measurement of a single diluted sample can be used to determine its optimal dilution factor.

## 1 Introduction

Extracellular vesicles (EVs) are lipid-membrane–enclosed particles that are released by all cells. As the biochemical composition, concentration, and function of EVs change in disease, EVs have biomarker potential [1].

Flow cytometry can be used to determine the concentration of EVs in blood plasma [2,3]. Flow cytometers measure light scattering and fluorescent signals from all particles, i.e., from EVs as well as non-EV particles. The light scattering signal provides information about the size of particles, and the fluorescence signal can provide information about the cell type of origin of EVs. The latter is achieved by staining EVs with fluorescently labeled and cell-type–specific antibodies [4]. A requirement for the detection of single particles is that their signal should exceed the threshold of light scatter and/or fluorescence detectors.

EVs in blood plasma are outnumbered by non-EV submicron particles, such as lipoproteins, proteins, and protein complexes. The size distribution of submicron particles in plasma steeply increases toward particles with a small diameter [[5], [6], [7]]. In other words, a bulk of small submicrometer particles is present in plasma that will not be detected as single particles by a flow cytometer, although the presence of such particles may still affect the measured signals. At sufficiently high concentrations of submicrometer particles, particles with signals below the trigger threshold of detectors are simultaneously and continuously illuminated by the laser beam [8]. When the combined signals of these particles exceed the trigger threshold, they will be measured as a single event. This special case of coincidence is called swarm detection and leads to incorrect measurements of EV concentrations [9,10].

To avoid swarm detection, samples require dilution. In the presence of swarm detection, the relation between the measured concentration of particles and the dilution factor starts to deviate from a linear function. In this article, we focus on blood plasma as EV-containing body fluid, but the approach for other body fluids and conditioned culture medium is comparable. Ideally, the dilution factor is kept to a minimum to maximize the number of measured particles. According to current guidelines of the American Heart Association, the optimal dilution factor should be determined by a dilution series for all samples [9,11].

**Figure 1:**
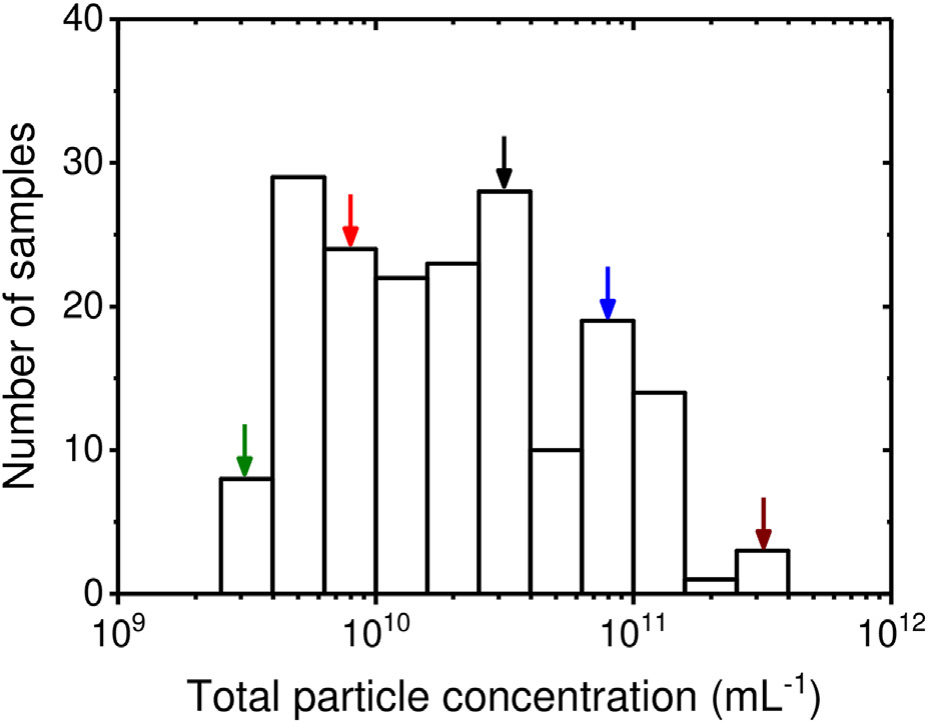
Distribution of the total particle concentration of samples from the clinical research study AFFECT EV (antiplatelet therapy effect on extracellular vesicles; *n* = 181). The concentration was determined by flow cytometry (Apogee A60-Micro) and reflects the number of particles exceeding a side scatter cross-section of 10 nm^2^. The concentrations differed 93-fold among these samples. The arrows indicate the samples that will be used in this study.

Figure 1 shows a distribution of the measured concentrations of submicrometer particles in plasma samples from a clinical study [12]. Since the measured total particle concentration differs by 2 orders of magnitude between these plasma samples, each sample requires its own optimal dilution factor. In daily practice, however, dilution series are laborious and incompatible with clinical routine. Hence, there is a need for a practical procedure to determine the optimal dilution factor for a series of clinical samples.

As explained, swarm detection is caused by particles below or at the detection limit of detectors, of which the concentration cannot be measured. Since the count rate is a measure of the number of particles present within a sample, especially those just above the detection limit, we hypothesize that the count rate of a flow cytometry measurement can be used to predict and, thus, avoid swarm detection. Therefore, we explored whether a fixed minimum dilution factor or maximum count rate can be used to prevent swarm detection. In addition, we measured the concentration and particle size distribution below the detection limit of the flow cytometer, to gain insight into the relationship between the presence of these particles and swarm detection.

## 2 Methods

### 2.1 Sample selection

Antiplatelet therapy effect on extracellular vesicles (AFFECT EV) was a randomized controlled trial that aimed to compare the effect of different antiplatelet drugs (P2Y12 receptor antagonists) on circulating plasma EV concentrations in patients with acute myocardial infarction (NCT02931045) [12,13]. Blood was collected from fasting patients at 3 different time points. Plasma was prepared using double centrifugation at 2500 *g* for 15 minutes and plasma aliquots were stored at −80 °C until EV analysis with flow cytometry. All details about patient inclusion, blood collection, sample handling, and storage can be found in references [12] and [13]. Figure 1 shows a histogram of the total particle concentrations, representing all particles exceeding a side scatter cross-section of 10 nm^2^. To systematically investigate swarm detection in samples with different particle concentrations, 5 plasma samples that differed ∼ $\sqrt{10}$ in total particle concentration were randomly selected, indicated with arrows in Figure 1. Samples were termed “donor 1” to “donor 5” and had a low (2.5 × 10^9^) to high (2.1 × 10^11^ mL^−1^) total particle concentration, respectively.

### 2.2 Sample preparation

Plasma samples were thawed in a water bath at 37 °C for 90 seconds. Dilution series were created by diluting samples in Dulbecco’s phosphate-buffered saline (DPBS). For labeling, 20 μL of diluted plasma was incubated with 2.5 μL of allophycocyanin (APC)-conjugated CD61, a cluster of differentiation that binds to platelet membrane glycoprotein, GPIIIa, and 2.5 μL of phycoerythrin-conjugated CD62p, which binds to P-selectin and kept in the dark at room temperature for 2 hours. To reduce the background fluorescence from unbound reagents, samples were further diluted by adding 200 μL of DPBS, resulting in an additional 11-fold dilution. Reported dilution factors indicate the total dilution factor during the measurement relative to plasma. The total dilutions for labeled samples ranged from 11 to 3.6 × 10^5^-fold, with increments of $\sqrt{10}$.

### 2.3 EV concentration measurements by flow cytometry

The total particle concentration and the concentration of platelet-derived EVs (PEVs; CD61^+^) were determined by flow cytometry (Apogee A60-Micro, Apogee Flow Systems). The flow rate was 3.01 μL∙min^−1^ and side scattering was used as trigger detector. The total particle concentration represents the number of particles per milliliter of plasma exceeding the side scattering cross-section of 6 nm^2^, which is 4 nm^2^ lower than that in clinical study AFFECT EV [13]. The particle count rate (hereafter: count rate) indicates the total number of particles that exceed the side scattering cross-section of 6 nm^2^, per second. The PEV concentration represents all particles that exceed the fluorescent gate of APC, which is 61 molecules of equivalent soluble fluorophore, and have a diameter <1000 nm (Rosetta Calibration v1.23, Exometry BV) per milliliter of plasma. Although CD62p was used to stain EVs derived from activated platelets, the obtained data had insignificant counts and were, therefore, not considered in the analysis.

### 2.4 Detection limit of flow cytometer

To achieve reproducible EV concentration measurements and to be able to compare our concentration measurements to those from other laboratories and instruments, the lower limit of detection (LoD) was determined for our flow cytometer and settings. To relate scatter signals to diameter of particles, Rosetta Calibration (version v1.23, Exometry) was used, which requires the particle refractive index (RI) as input. We assumed that lipoproteins are solid particles that have an RI of 1.47 [14], while EVs were assumed to have a core RI of 1.38, a shell thickness of 6 nm, and a shell RI of 1.48 [15]. The LoD of flow cytometry, in terms of particle diameter, was determined by taking the mode of the particle size distribution (PSD) in FlowJo (v10, FlowJo). This analysis resulted in an LoD of 110 nm for lipoproteins and 146 nm for EVs.

### 2.5 Data outliers

Data obtained for the 5 samples measured at 10 different dilutions are based on single measurements of labeled samples. However, the count rate of the 3.6 × 10^3^-fold dilution of the plasma from donor 2 was similar to the count rate of the background measurements and was therefore excluded from analysis. Instead, for this measurement of donor 2, the total particle concentration and count rate were derived from the measurement of the same unlabeled sample, which had a dilution factor of 3.5 × 10^3^-fold. Even though the measurement of the 1.1 × 10^4^-fold dilution of donor 5 had a stable count rate, it resulted in a higher concentration than anticipated and was therefore repeated. For this duplicate measurement, the error bars represent the lower and higher concentrations, whereas the symbol represents the average value.

The 11-fold diluted sample of donor 5 could not be measured because the particle concentration and count rate were too high to be handled by our flow cytometer electronics. The measurement of the 1.1 × 10^5^-fold diluted sample of donor 1 failed and was therefore excluded from analysis.

### 2.6 Data analysis

Custom-built software (MATLAB R2020b, MathWorks) was used for data calibration and analysis. Linear functions were fitted to the data by least square fitting. For count rate vs dilution plots, the slope of the linear function was fixed at −1. For concentration vs count rate plots and concentration vs dilution plots, the slope of the linear function was fixed at 0. In the presence of swarm detection, the relation between the measured concentration of particles and the dilution factor starts to deviate from a linear function. Data points that deviated <20% from the fit line were considered reliable, ie, without swarm detection. The experiments fulfill the standardized reporting framework MIFlowCyt-EV [16,17].

### 2.7 PSD by microfluidic resistive pulse sensing

Microfluidic resistive pulse sensing (MRPS; nCS1, Spectradyne) was used to measure the PSD, which we define as the concentration of particles vs their diameter, in plasma. Samples were diluted 75- to 150-fold in DPBS with 0.1% bovine serum albumin. Thereafter, 5 μL of sample was measured using both C400 (specified size range, 65-400 nm) and C2000 (specified size range, 250-2000 nm) cartridges. The LoD of MRPS using C400 cartridges was previously determined at 75 nm [18]. nCS1 software (version 2.5.0.297, Spectradyne) was used for data analysis.

The first data point of the size distributions measured with both C400 and C2000 cartridges is unreliable and was therefore excluded [18]. The specified detection ranges of C400 and C2000 cartridges overlap between 265 nm and 400 nm. Within this size range, the Poisson error of the concentrations measured with C400 cartridges exceeds that of the concentrations measured with C2000 cartridges. Hence, data from 70 nm to 260 nm were acquired with C400 cartridges, whereas data ≥260 nm were obtained with C2000 cartridges. Next, the data were fitted with a sum of 2 power-law functions, indicating the distributions for both lipoproteins and EVs. To provide an indication of the PSDs during the flow cytometry measurements, the obtained fits were divided by dilution factors that were used during flow cytometry measurements. The corresponding values of the fit parameters, coefficients of determination (R^2^), as well as the individual data points, can be found in the Supplementary Material, Supplementary Table 1 and Supplementary Figure 1.

Since our flow cytometer has an LoD of 110 nm for lipoproteins, particles with a diameter <110 nm are only detected if their combined signals exceed the scattering threshold, i.e., when swarm detection occurs. Therefore, we compared the fitted MRPS data with the maximum concentrations of particles <110 nm that would prevent swarm detection. Rosetta Calibration (v2.00, Exometry) was used to determine the number of particles required to obtain a scattering cross-section similar to that of a single 110-nm particle, resulting in a maximum PSD <110 nm that would avoid swarm detection. The maximum particle concentration at 110 nm in the absence of swarm detection was used as reference.

## 3 Results

### 3.1 Dilution and count rate as swarm detection criteria

**Figure 2:**
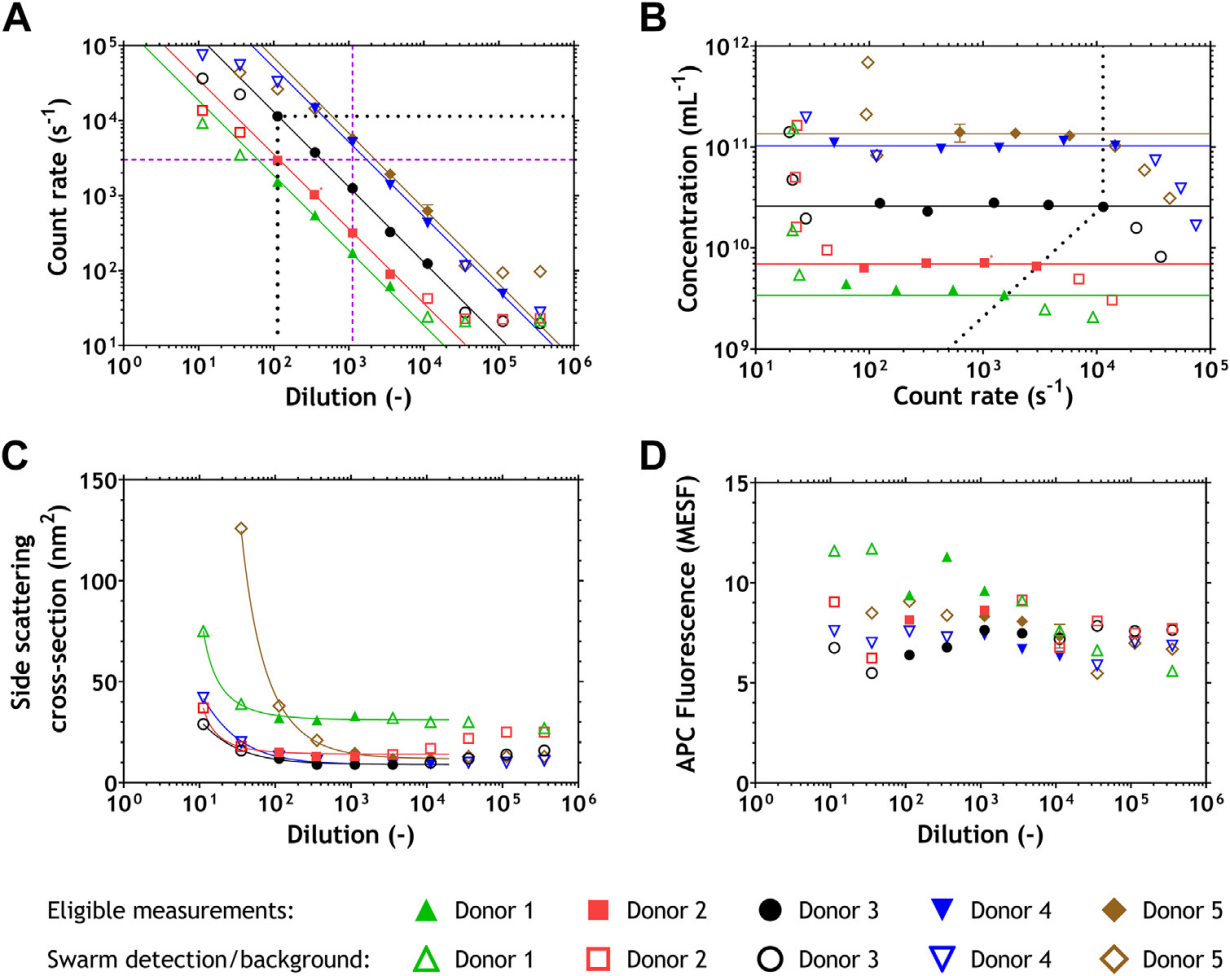
Flow cytometry data of 5 selected samples, indicating particles exceeding a side scattering cross-section of 6 nm^2^. In panels A and B, data were fitted on the first 3 data points that exceed a count rate of 1.5 × 10^2^ events·s^−1^. Open symbols resemble data points that deviate >20% from the fit lines. (A) Count rate (events·s^−1^) plotted vs the dilution factor, fitted with a linear function (lines; slope, −1; R^2^, 0.94, 0.94, 0.99, 0.99, and 0.97 for donors 1 to 5, respectively). Open data points on the left are affected by swarm detection, while those on the right are attributed to background counts. Purple dashed lines visualize a count rate of 3.0 ×·10^3^ events·s^−1^ (horizontal) and a dilution of 1.1 × 10^3^-fold (vertical). Measurements exceeding a dilution of 1.1 × 10^2^-fold and below a count rate of 1.1 × 10^4^ events·s^−1^ are unaffected by swarm detection (black dotted line). (B) Total particle concentration plotted vs the count rate (events·s^−1^), fitted with a horizontal line. Open symbols on the left are attributed to background events (<1.5 × 10^2^ events·s^−1^), while open data points on the right area are affected by swarm detection. The black dotted line represents a 1.1 × 10^2^-fold dilution and a count rate of 1.1 × 10^4^ events·s^−1^ and distinguishes eligible measurements from those affected by swarm detection. (C) Median side scattering cross-section (nm^2^) plotted vs the dilution factor, fitted with a reciprocal function (+ offset) on data points <2 × 10^4^-fold diluted. Open symbols do not fulfill the defined criteria represented by the black lines in panels A and B. Measurements of donor 1 were performed in another time frame than the measurements of donors 2 to 5, thereby affecting the median side scattering cross-section. (D) Median fluorescence on the APC detector plotted vs the dilution factor. APC, allophycocyanin; MESF, molecules of equivalent soluble fluorophore.

To evaluate whether dilution factor and/or count rate are indicators of swarm detection, Figure 2A shows the count rate vs dilution of the 5 plasma samples. In the absence of swarm detection, the count rate decreases linearly with increasing dilution. The solid lines are a linear fit of the first 3 data points exceeding the background events, which are considered measurements with count rates <1.5 × 10^2^ events·s^−1^. At the lowest dilutions, most count rates fall below the fits due to swarm detection. At the highest dilutions, the count rates exceed the linear fits because background events dominate the count rate. At a 1.1 × 10^3^-fold dilution, swarm detection is absent in all plasma samples (vertical purple dashed line), but at this dilution, 4 out of 5 samples are measured at an unnecessarily high dilution, which means a longer measurement time (e.g., 200 minutes for donor 1) is required to obtain a sufficient number of particle counts. Thus, a fixed dilution is unsuitable to avoid swarm detection when aiming to maximize particle and EV counts.

Figure 2A also shows that the maximum count rate without swarm detection is 3.0 × 10^3^ events·s^−1^ (horizontal purple dashed line). However, a count rate of 3.0 × 10^3^ events·s^−1^ would mean an unnecessarily high dilution for 3 samples. Therefore, a fixed count rate is also an unsuitable approach when aiming to maximize EV counts. Nevertheless, swarm detection is prevented for all donors when a minimum dilution factor of 1.1 × 10^2^-fold is combined with a maximum count rate of 1.1 × 10^4^ events·s^−1^ (dotted lines). Thus, the optimal approach to prevent swarm detection is to use a combination of the dilution factor and count rate.

### 3.2 Validation of swarm detection criteria

To validate whether a minimum dilution factor of 1.1 × 10^2^-fold and a count rate below 1.1 × 10^4^ events·s^−1^ prevent swarm detection in plasma samples measured on our flow cytometer, Figure 2B shows the total measured particle concentrations in plasma vs the count rate. Ideally, the relationship between the measured particle concentration and count rate is linear. Therefore, the solid lines in Figure 2B represent a horizontal fit of the first 3 data points exceeding the background count rate of 1.5 × 10^2^ events·s^−1^. At low count rates (left), background events dominate and lead to overestimation of the measured concentration. At high count rates (right), swarm detection results in underestimation of the measured particle concentration. Figure 2B shows that all data fulfilling the swarm detection criteria (dotted lines) defined in Figure 2A fall onto the linear fit, thereby confirming that the criteria are valid.

Scattering or fluorescence signals may potentially be used to indicate the presence of swarm detection when running dilution series. Figure 2C shows the median side scattering cross-section, which was fitted with a reciprocal function for dilutions ≤2.0 × 10^4^-fold. Data points that fulfill the defined criteria equal the offset of the reciprocal function, indicating that the median side scattering cross-section was stable for these measurements. At low dilutions, the scattering cross-section was substantially increased. The median side scattering cross-section can thus be used to indicate swarm detection when analyzing dilution series.

Figure 2D shows the median fluorescence signals as detected on the APC detector. All 5 samples show a median APC fluorescence of 8 ± 3 molecules of equivalent soluble fluorophore, indicating that the median fluorescence of all particles does not clearly indicate swarm detection.

### 3.3 Evaluation of defined criteria on platelet EVs

**Figure 3:**
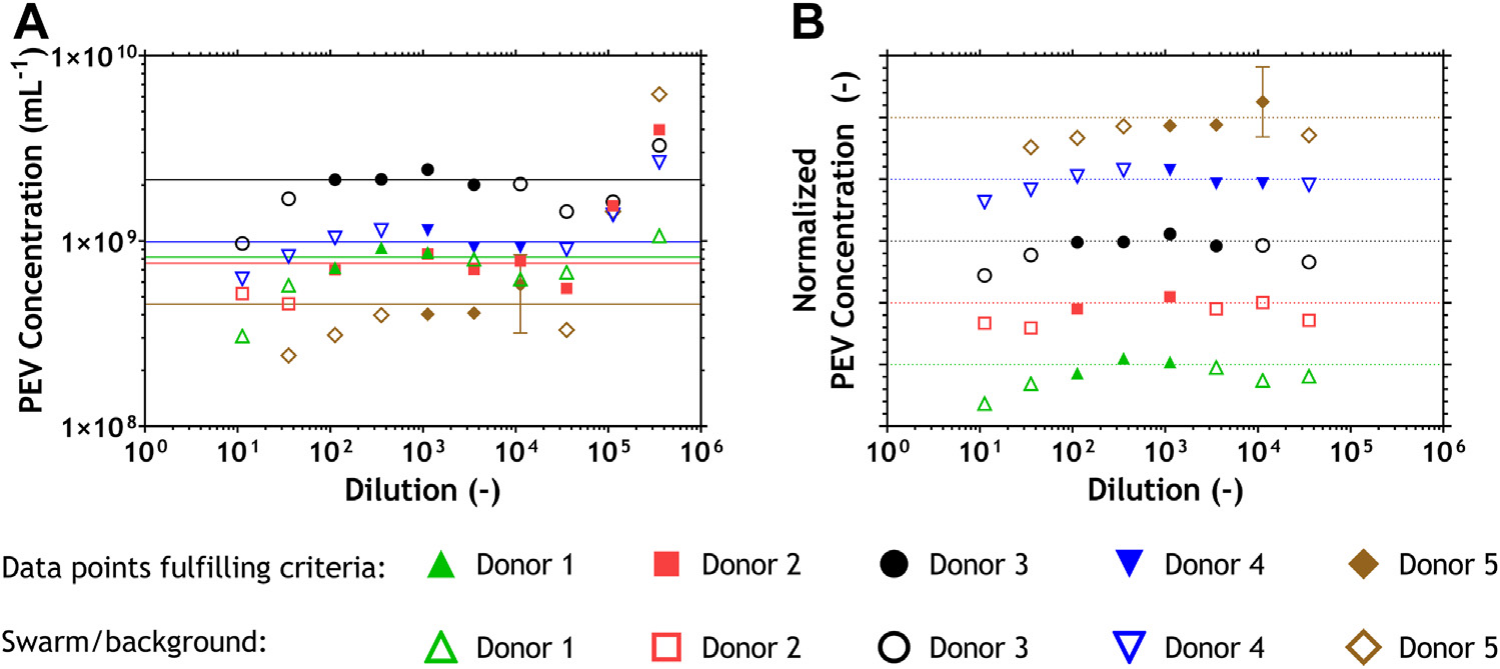
Flow cytometry data of 5 selected samples, indicating particles exceeding a side scatter cross-section of 6 nm^2^, having a diameter <1000 nm, and exceeding 61 molecules of equivalent soluble fluorophore for CD61-APC–labeled particles. Filled data points meet the criteria of being diluted ≥1.1 × 10^2^-fold and having a count rate below 1.1 × 10^4^ events·s^−1^. (A) PEV concentration vs dilution, in which the filled data points were fitted with a horizontal line. (B) Measured PEV concentration normalized to the mean concentration derived from the fit in panel A and plotted vs the dilution. Data of each donor are separated with an offset of 1. Horizontal lines indicate a ratio of 1, meaning that the measured concentration equals the mean concentration, while minor tick marks indicate a 20% deviation from the mean concentration. APC, allophycocyanin; PEV, platelet-derived extracellular vesicle.

To evaluate whether the previously defined criteria, i.e., a dilution factor ≥1.1 × 10^2^-fold and a count rate <1.1 × 10^4^ events·s^−1^, also lead to reliable concentration measurements of PEVs, Figure 3A shows the PEV concentrations measured at different dilutions. When the PEV concentration is independent of measurement dilution, there is no swarm detection. The filled data points, i.e., PEV concentrations that fulfill the previously defined criteria to prevent swarm detection, can be fitted with a linear function. The mean concentration of PEVs could be derived from the fit parameters.

Figure 3B shows the concentration of PEVs relative to the mean concentration of PEVs. To prevent overlap between data points from different donors, the normalized concentrations have an offset of 1, and data acquired at a dilution factor ≥10^5^-fold were excluded. Again, filled data points fulfill the criteria that prevent swarm detection. which are defined in Figure 2A. All filled data points, except the one with the error bar, deviate <20% from the mean concentration. Thus, a minimum dilution factor of 1.1 × 10^2^-fold and a maximum count rate of 1.1 × 10^4^ events·s^−1^ lead to EV concentration measurements with <20% deviation from the calculated mean.

### 3.4 PSD by MRPS

**Figure 4:**
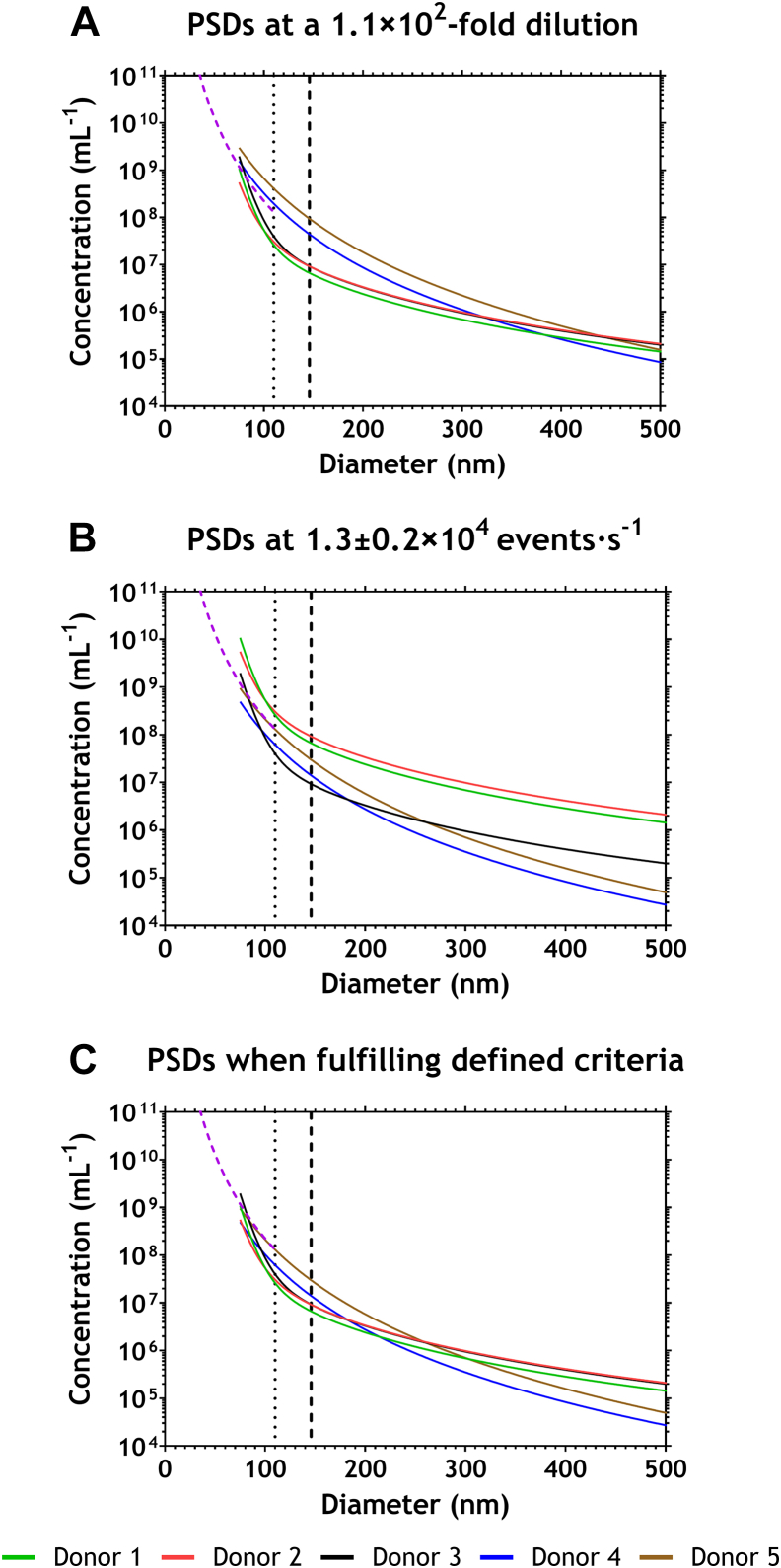
Microfluidic resistive pulse sensing data of selected samples visualizing the fits of the particle size distribution (PSD) with a bin width of 10 nm. Data were acquired at a single dilution and fitted with a third-order polynomial function; fitted data were then adjusted for dilution factors used during the flow cytometry experiment. Plots start from 75 nm, which is the lower limit of detection (LoD) for the microfluidic resistive pulse sensing measurements. The LoD for the flow cytometer is indicated with dotted lines for lipoproteins (110 nm) and with dashed lines for extracellular vesicles (146 nm). The purple, dashed diagonal line indicates the particle concentrations below the LoD of the flow cytometer needed to trigger swarm detection. (A) PSDs at a total dilution of 1.1 × 10^2^-fold for all samples. (B) PSDs at a count rate of 1.3 ± 0.2 × 10^4^ events·s^−1^, representing total dilutions of 11-fold for donors 1 and 2, 1.1 × 10^2^-fold for donor 3, and 3.6 × 10^2^-fold for donors 4 and 5. (C) PSDs when samples fulfill defined criteria to prevent swarm detection, representing a total dilution of 1.1 × 10^2^-fold for donors 1, 2, and 3 and 3.6 × 10^2^-fold (count rate, 1.3 ± 0.2 × 10^4^ events·s^−1^) for donors 4 and 5.

To explain why our criteria prevent swarm detection, we used MRPS to measure the PSDs of plasma samples because MRPS can also detect particles below the LoD of the flow cytometer. For each of the previously described criteria to prevent swarm detection, Figure 4 shows the calculated PSDs during the flow cytometry measurements combined with the maximum PSD <110 nm that would prevent swarm detection (purple dashed line).

Figure 4A shows that when the plasma samples were 1.1 × 10^2^-fold diluted, the particle concentrations of donors 4 and 5 were substantially higher than those of the other 3 donors, especially in the size region below the LoD of the flow cytometer, which is ≥110 nm for lipoproteins (black dotted line) and ≥146 nm for EVs (black dashed line). The PSDs of donors 4 and 5 also exceed the determined maximum PSD for particles <110 nm that would avoid swarm detection. Thus, the MRPS data support that 1.1 × 10^2^-fold dilution is sufficient to avoid swarm detection in samples from donors 1, 2, and 3 but not in those from donors 4 and 5 (Figure 2A).

Figure 4B shows PSDs resembling samples that had been diluted to realize a count rate of 1.3 ± 0.2 × 10^4^ events·s^−1^. Across the entire size range, the particle concentrations of donors 1 and 2 are substantially higher than those of the other 3 donors. The PSDs of donors 1 and 2 also exceed the determined maximum PSD for particles <110 nm. Thus, the MRPS data support that a count rate of 1.1 × 10^4^ events·s^−1^ on the flow cytometer prevents swarm detection in samples from donors 3, 4, and 5, but a lower count rate is required for samples from donors 1 and 2.

Figure 4C shows that the PSDs of all 5 samples overlap when they meet the criteria of a ≥1.1 × 10^2^-fold dilution and a count rate <1.1 × 10^4^ events·s^−1^. In sum, Figure 4 shows that for all measurements without swarm detection, the concentrations of particles below the LoDs of flow cytometry are below the dashed purple line. This line indicates the particle concentrations per bin width of 10 nm for particles <110 nm that should be exceeded to meet the triggering threshold for our flow cytometer, thereby resulting in swarm detection. Our previously defined criteria to prevent swarm detection ensure that the PSDs fall below the dashed purple line and the LoD of flow cytometer.

## 4 Discussion

Reproducible concentration measurements of EVs are required to utilize EVs as blood-based biomarkers and introduce EVs into clinical practice. At present, EV flow cytometry requires dilution series for all samples to determine the optimal dilution factor that prevents swarm detection [9,10,16]. However, performing extensive dilution series for all samples is unfeasible in clinical routine. Therefore, we aimed to develop a practical procedure to prevent swarm detection while minimizing the dilution factor, so that particle counts are maximized.

To establish a procedure that prevents swarm detection, we looked at dilution and count rate because these parameters can either be controlled by the operator (dilution) or measured (count rate). When measuring blood plasma with our flow cytometer, the optimal approach to prevent swarm detection is a minimum dilution factor of 1.1 × 10^2^-fold combined with a maximum count rate of 1.1 × 10^4^ events·s^−1^. Independent measurements of the PSDs of the plasma samples indicated that below the LoD of the flow cytometer, particle concentrations should be sufficiently high to trigger swarm detection. When the defined criteria are met, particle concentrations below the LoD of the flow cytometer are not high enough to trigger swarm detection. To find the optimal dilution factor of human plasma for our flow cytometer and settings, we recommend to 1) measure the count rate of a single highly (1.0 × 10^3^-fold) diluted sample and 2) calculate the dilution factor to achieve a count rate of <1.1 × 10^4^ events·s^−1^ while requiring a minimum dilution of 1.1 × 10^2^-fold.

Requiring a minimum dilution factor and a maximum count rate does not support our hypothesis that only the count rate of a flow cytometer is indicative of swarm detection. Our hypothesis was based on knowledge of the size distribution of particles in body fluids measured by flow cytometry, which typically follows a power-law or exponential decaying function [19]. Consequently, the count rate is 1) dominated by particles that just exceed the LoD and 2) assumed to be predictive of the concentration of particles below the LoD, which may cause swarm detection. However, Figure 4B shows the size distribution of submicrometer particles in blood plasma follows a function composed of the sum of 2 power-law functions, most likely reflecting the contribution of both lipoproteins and EVs separately.

The variation in the particle concentration and size distribution is thus challenging EV flow cytometry studies. Both the particle concentration and the size distribution may be measured with a technique that can measure particles below the LoD of the flow cytometer, as we did with MRPS (Figure 4). The measured concentration and size distribution together with the fluidics and optical configuration of the flow cytometer could in principle be used to predict the dilution at which swarm detection is absent. However, reliable measurements of the concentration and size distribution below the LoD of a flow cytometer are laborious and costly. Whereas we used MRPS to measure the concentration and size distribution, nanoparticle tracking analysis (NTA) is by far the most popular technique to measure size distributions of EV-containing samples [20]. However, NTA is substantially less accurate and precise compared to MRPS in determining the diameter and concentration [19]. Based on the performance of NTA and the subtle differences in size and concentration that are indicative of swarm detection (Figure 4), we anticipate that NTA is unsuitable to predict a dilution factor that prevents swarm detection. In sum, a practical approach to prevent swarm detection in EV flow cytometry is the application of a minimum dilution factor and a maximum count rate. The presented methodology can be used to derive these criteria for other sample types, flow cytometers, and settings. Once these criteria are known, it takes only a single measurement instead of an entire dilution series to obtain the optimal dilution to prevent swarm detection. Therefore, we estimate a time saving of 10 to 15 minutes per sample. Moreover, since each sample is eventually measured twice, i.e., once overdiluted to determine the optimal dilution factor and once at that optimal dilution, it is possible to check whether swarm detection is indeed prevented. Without swarm detection, the measured total particle concentrations of these 2 measurements should be similar.

### 4.1 Limitations

The findings of this study have to be considered in the light of potential limitations being 1) the investigation of scatter triggering only, 2) the use of 1 flow cytometer, and 3) the sample size.

First, because all particles scatter light, this research focused on scatter-triggered flow cytometry measurements. In case of fluorescence detection, only labeled particles and unbound fluorophores emit fluorescence (with the exception of relatively dim autofluorescence). Swarm detection, therefore, increases the scatter signals of all events but not necessarily the fluorescence signals of all events. In addition, the total counts of immunofluorescently labeled EVs in plasma are typically too low to use for any predictions about swarm detection, as we experimentally confirmed. In Figure 2C, we showed that for (side) scatter-triggered measurements, the median side scattering cross-section of all particles and PEVs only (Supplementary Figure 2) are indicative of swarm detection in a dilution series. On the other hand, Figure 2D shows that the median fluorescence of plasma stained with CD61-APC is not indicative of swarm detection. Hence, to prevent swarm detection for fluorescence-triggered measurements, a new approach needs to be established [10]. For some flow cytometers, light scatter detection and triggering results in 15- to 75-fold lower concentration estimates compared to fluorescence triggering of the same EV-containing sample [21]. However, our flow cytometer has a high sensitivity on scatter and measures comparable concentrations for both fluorescence and scatter-based triggering [22].

Second, a single flow cytometer was used for this research. To extend our results to other flow cytometers, similar experiments should be repeated to investigate instrument-specific effects. We anticipate that similar criteria can be defined, resulting in specific numbers concerning the minimum dilution and/or maximum count rate, which was confirmed by the successful application of our proposed procedure to a second flow cytometer. As the flow rate, interrogation volume, and sensitivity affect the count rate and minimum dilution factor at which swarm detection occurs, the exact count rate and dilution factor are instrument-dependent. For example, reducing the sample flow rate decreases the sample stream width and the interrogation volume and hence decreases the concentration of particles being simultaneously present within the focus of the laser [8]. In a dilution series measured at 5 different flow rates, varying from 0.75 to 6.0 μL·min^−1^, we noticed that the dilution required to prevent swarm detection scales proportionally with the flow rate. We expect that this finding also applies to other flow cytometers. The maximum count rate at which swarm detection occurs is independent of the flow rate (see Supplementary Material, Supplementary Figure 3). Therefore, in our experiments, we limited ourselves to our commonly used flow rate of 3.01 μL·min^−1^.

Third, we selected plasma samples with varying total particle concentrations. As these samples may not be representative of all (plasma) samples, we advise investigating swarm detection in samples outside the investigated range of concentrations. We recommend finding the minimum dilution factor and maximum count rate for each sample type separately, such as for biological fluids other than blood plasma, cell lines, or freshly prepared plasma samples. Apart from the total particle concentration, the particle concentration just below the detection limit of the flow cytometer or the slope of the PSD might be variables affecting swarm detection. An experiment to investigate how variables other than the total particle concentration might affect swarm detection would involve 1) measuring these variables in many samples, including biological fluids other than blood plasma, cell lines, or freshly prepared plasma samples; 2) making a selection of samples to perform systematic research; and 3) performing dilution series. From a practical viewpoint, given the combinations of the possible variables involved and the difficulty in measuring them, it is almost infeasible to conduct such an experiment. Therefore, we are currently developing a simulation of a flow cytometer to systematically investigate sample- and flow cytometer–related variables involved in swarm detection.

## 5 Conclusion

We showed that combining a minimum dilution factor with a maximum count rate prevents swarm detection in EV flow cytometer while ensuring sufficient particle counts. Thus, the count rate of a single overdiluted sample is sufficient to calculate the optimal dilution factor for that particular sample, thereby ensuring that the criteria of minimum dilution factor and maximum count rate are met. This procedure is fast and practical and ensures reliable EV flow cytometry concentration measurements. The presented methodology can also be applied to prevent swarm detection in other sample types, on flow cytometers other than ours, and using different settings. The use of such procedures will increase the chances of bringing EVs into clinical practice.

## Supplementary material

Supplementary Figures Tables

MIFlowCyt-EV_Preventing_swarm_detection
