# Supplementary material for: Preventing swarm detection in extracellular vesicle flow cytometry: a clinically applicable procedure
Source: Res Pract Thromb Haemost. 2023 May 2;7(4):100171. doi: 10.1016/j.rpth.2023.100171 (PMC10239693; doi:10.1016/j.rpth.2023.100171)
Supplement: Supplementary Figures — Tables [file mmc1.pdf]

Supplementary Material of "Preventing swarm detection in extracellular vesicle flow cytometry: a clinically applicable procedure"

Raw Microfluidic Resistive Pulse Sensing (MRPS) data

The concentration (C) and diameter (d) measured by MRPS were fitted with a combination of two power-law functions:

$$C = ad^b + fd^g$$

where C is the concentration as a function of diameter d, and a, b, f, and g are variables. The sum of two power-law functions was chosen, since it is assumed these samples consist mainly of lipoproteins and EVs, but their effect on the PSD may depend on the ratio between the concentrations of these particles. Supplementary Table 1 contains the fit parameter values of all five samples, as well as the coefficient of determination (R^2). Supplementary Figure 1 shows the fits combined with the original MRPS data from both the C400 and C2000 cartridge, with 10 nm bin width.

Supplementary Table 1. Fit parameter values of the sum of power-law functions, used to fit the microfluidic resistive pulse sensing data.

	a	b	f	g	R^2
Donor 1	$-1.9 \cdot 10^{33}$	$-1.2 \cdot 10^{1}$	$3.1 \cdot 10^{15}$	-3.1	0.999
Donor 2	$4.4 \cdot 10^{29}$	$-1.0 \cdot 10^{1}$	$3.2 \cdot 10^{15}$	-3.0	0.999
Donor 3	$5.9 \cdot 10^{33}$	$-1.2 \cdot 10^{1}$	$3.9 \cdot 10^{15}$	-3.1	0.995
Donor 4	$7.6 \cdot 10^{22}$	-6.4	$1.8 \cdot 10^{20}$	-4.9	0.741
Donor 5	$1.1 \cdot 10^{21}$	-5.2	$7.4 \cdot 10^{20}$	-5.2	0.992

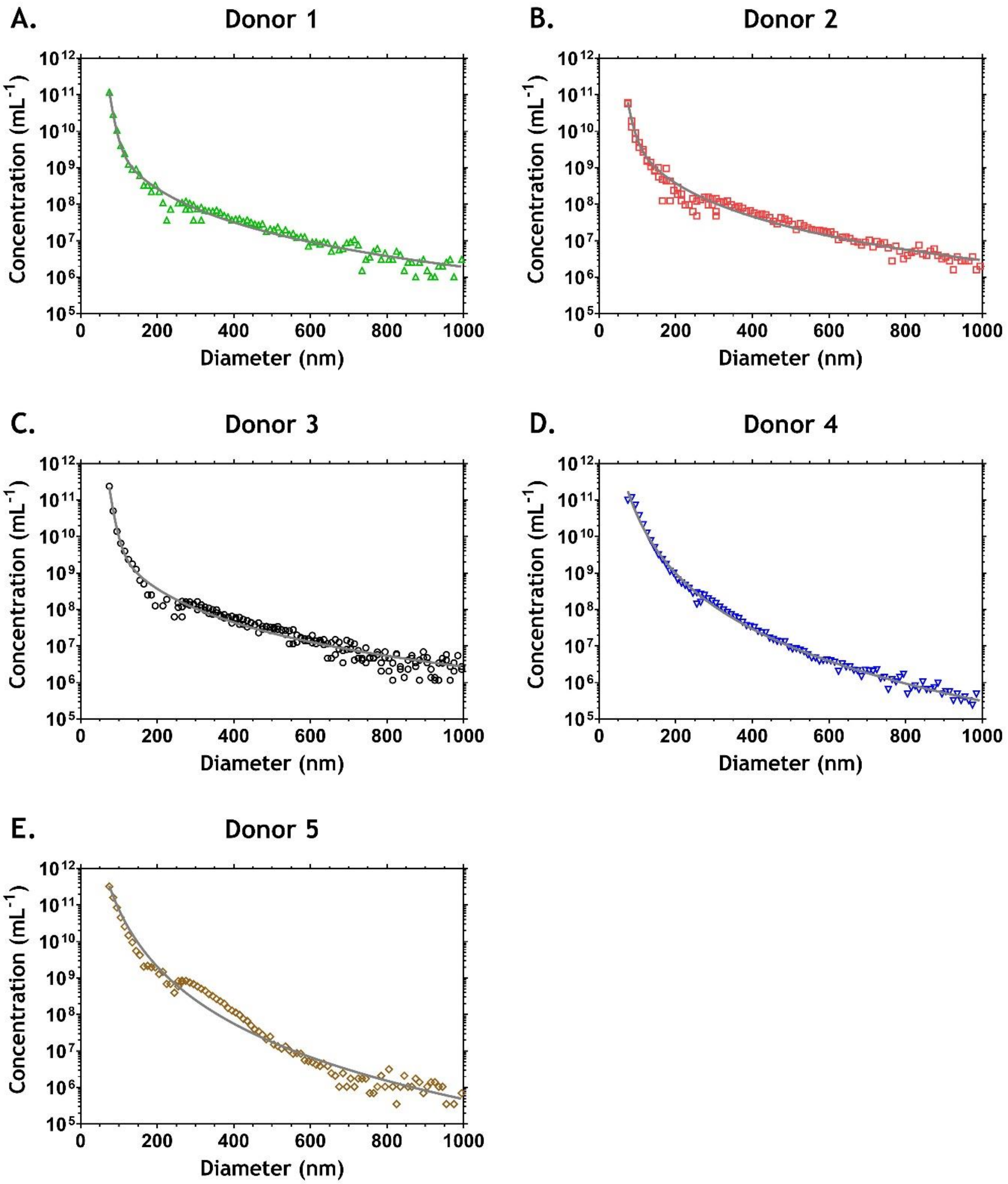

Supplementary Figure 1. Microfluidic Resistive Pulse Sensing (MRPS) data of selected samples, visualizing the particle size distribution (PSD) with a bin width of 10 nm. Data in figures A to E represent the PSDs for undiluted samples of donors 1 to 5, respectively. The grey lines indicate the fit, based on the sum of two power-law functions, which has also been used to visualize the PSDs in Figure 4.

Side scattering cross-section in situations with and without swarm detection

In Supplementary Figure 2, the distribution of the side scatter cross-section for platelet-derived EVs (PEVs) was plotted for four different dilutions of donor 3. For $1.1 \cdot 10^2$- and $3.6 \cdot 10^2$-fold diluted samples, swarm detection was absent and the distributions overlap. At these dilutions, the side scatter cross-section shows a sharper response function close to the lower limit of detection (LoD) than lower diluted samples. For $1.1 \cdot 10^1$- and $3.6 \cdot 10^1$-fold diluted samples, swarm detection was present and (1) the measured concentration decreases compared to samples without swarm detection and (2) the distributions of the side scatter cross-section show a broad response function close to the LoD. We expect that under conditions of swarm detection the detector response function close to the LoD broadens due to increased background and background noise levels.

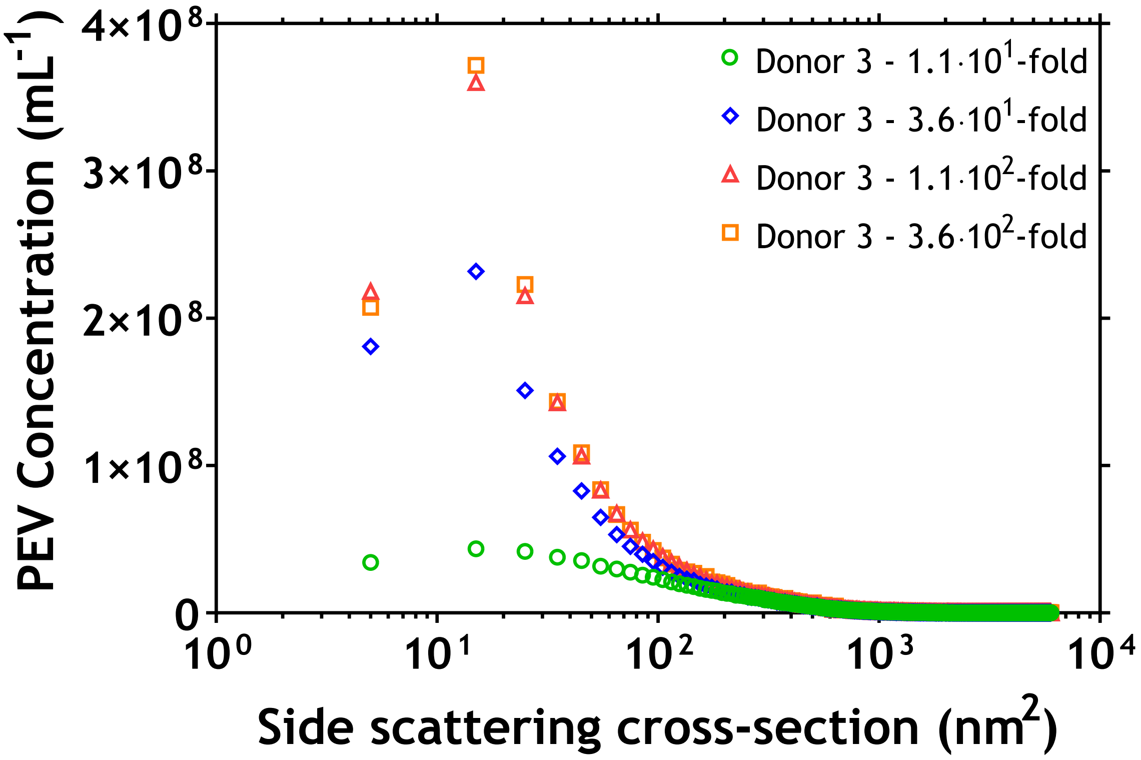

Supplementary Figure 2. Distribution of the side scattering cross-section for PEVs. PEVs were identified with flow cytometry and represent those particles exceeding a side scatter cross-section of 6 nm^2, having a diameter <1,000 nm and exceeding 61 molecules of equivalent soluble fluorophore for allophycocyanin-conjugated CD61.

Swarm detection at different flow rates

Flow cytometers are originally used to detect cells and do so with flow rates between 10 and 50 $\mu L \cdot min^{-1}$ (Kuiper, van de Nes, Nieuwland, Varga, & van der Pol, 2021). In EV flow cytometry, flow rates are reduced to decrease the sample stream width and thereby the interrogation volume. Since this would decrease the probability of multiple particles being simultaneously present within the focus of the laser, we initially ran a dilution series at five different flow rates. Supplementary Figure 3A shows the median side scattering cross section of a sample when measured at different dilution factors ($8 \cdot 10^0$- to $1.6 \cdot 10^4$-fold) and at different flow rates (0.75 to 6.0 $\mu L \cdot min^{-1}$). At dilutions $\geq 2.6 \cdot 10^2$, the median side scattering cross section is stable for all five flow rates. However, based on the median side scattering cross section, one could also use a dilution of $3.2 \cdot 10^1$-fold when measuring at the lowest flow rate. However, Supplementary Figure 2B shows that the flow rate does not affect the particle count rate for which swarm detection occurred. At all flow rates, the concentration estimates are affected by swarm detection for count rates $\geq 1.1 \cdot 10^4$ $events \cdot s^{-1}$.

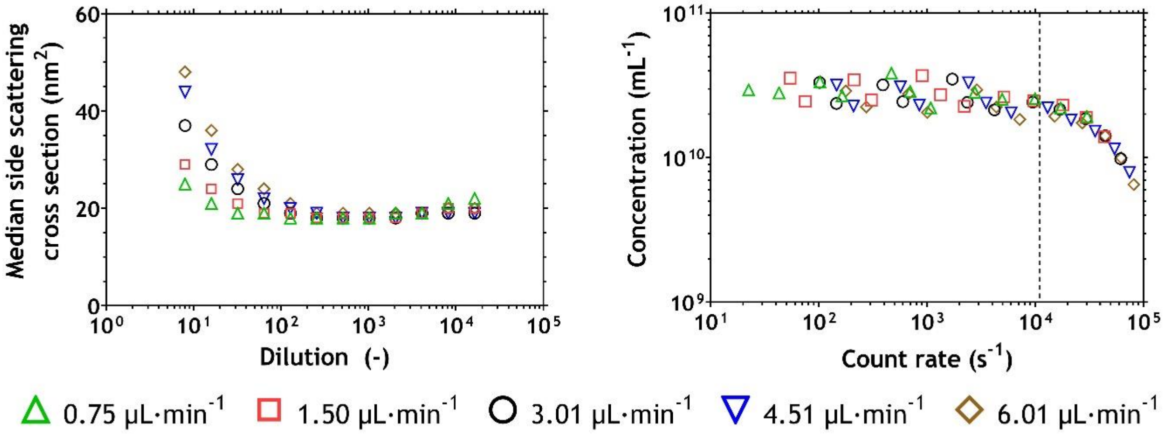

Supplementary Figure 3. Flow cytometry data of one sample of pooled plasma from healthy volunteers, indicating particles exceeding a side scatter cross section of 10 nm². Samples were measured at 5 different flow rates. (A) Median side scattering cross section (nm²) of all particles versus dilution. An increase in the median side scattering cross section indicates swarm detection. (B) The total particle concentration versus the count rate (events·s⁻¹). The black dotted line represents a count rate of 1.1·10⁴ events·s⁻¹.

Kuiper, M., van de Nes, A., Nieuwland, R., Varga, Z., & van der Pol, E. (2021). Reliable measurements of extracellular vesicles by clinical flow cytometry. *Am J Reprod Immunol, 85*(2), e13350. doi:10.1111/aji.13350
