# Supplementary material for: Preventing swarm detection in extracellular vesicle flow cytometry: a clinically applicable procedure
Source: Res Pract Thromb Haemost. 2023 May 2;7(4):100171. doi: 10.1016/j.rpth.2023.100171 (PMC10239693; doi:10.1016/j.rpth.2023.100171)
Supplement: MIFlowCyt-EV_Preventing_swarm_detection [file mmc2.pdf]

MIFlowCyt-EV of “Preventing swarm detection in extracellular vesicle flow cytometry - a clinically applicable procedure”

1 Flow cytometry

1.1 Experimental design

The aim of flow cytometry (A60-Micro, Apogee Flow Systems, Hemel Hempstead, UK) experiments was to define criteria that would prevent swarm detection in plasma samples. The total particle concentrations as well as the concentrations of extracellular vesicles (EVs) released from platelets (CD61$^+$) or activated platelets (CD62p$^+$) were measured in blood plasma samples to investigate for which criteria these concentrations were stable. We hypothesized that a either a fixed dilution or a maximum count rate could be used to prevent swarm detection in flow cytometry measurements of plasma samples.

All samples were measured using an autosampler, which facilitates subsequent measurements of samples in a 96-well plate. For this study, samples were measured on four different days (three in August - October 2021, one in September 2022). Each day a buffer-only control was measured as well as antibody in buffer controls and isotype controls corresponding to the labels in the well plate. Flow rate and scatter calibrations were validated daily. Fluorescence calibration was performed twice during this time period. To automatically determine optimal sample dilutions, apply calibrations, determine and apply gates, generate reports with scatter plots and generate data summaries, we used custom-build software (MATLAB R2020b, Mathworks, Natick, MA, USA).

1.2 Sample dilutions

We measured serial dilutions of blood plasma from 5 patients. Prior to staining, dilutions ranged from undiluted to $3.2 \cdot 10^5$-fold diluted. Post-staining (see section 1.3), the final dilutions ranged from 11.25- to $3.6 \cdot 10^6$-fold. Count rates varied from 19.85 events$\cdot$s^{-1} for the most diluted to 74,626 events$\cdot$s^{-1} for the most concentrated samples.

1.3 EV staining

EVs were stained with antibodies. Prior to staining, antibodies were diluted in DPBS and centrifuged at 18,890 g for 5 min to remove aggregates. Table S1 shows an overview of the used reagents and antibody concentrations during staining. To stain, 20 µL of pre-diluted plasma was incubated with 2.5 µL of antibodies or isotype controls and kept in the dark

for 2 h at room temperature. Post-staining, samples were diluted 11.25-fold in 200 µL of DPBS to decrease background fluorescence from unbound reagents.

1.4 Buffer-only control

Each measurement day at least 1 well with DPBS was measured with the same flow cytometer and acquisition settings as the samples. The mean count rate was 21.4 events·s^{-1}.

1.5 Buffer with reagents control

Each measurement day, a buffer with reagent control for each reagent (Table S1) was measured with the same flow cytometer and acquisition settings as the samples. The mean count rate was 19.4 and 14.1 events·s^{-1} for buffer with CD61-APC or CD62p-PE controls, respectively, which is similar to the buffer-only control.

1.6 Unstained controls

For a selection of the samples, unstained controls were measured with the same flow cytometer and acquisition settings as the stained samples. The count rates of these measurements were in the same range as those of the stained samples.

1.7 Isotype controls

Table S1 shows an overview of the used isotype controls, which were added to a selection of samples. For 5 plasma control samples, we obtained an average of 21.2 IgG1-APC^{+} events and 11.8 IgG1-PE^{+} events with a diameter ≤1,000 nm per measurement. For comparison, using the same sample dilutions, on average 23957 CD61-APC^{+} events and 583.4 CD62p-PE^{+} events with a diameter ≤1,000 nm were obtained in the experiments using plasma samples.

1.8 Trigger channel and threshold

The optimal SSC voltage has been found and set using regular voltration. The background noise on the SSC detector was recorded while measuring a buffer-only control triggering on the background noise of an irrelevant fluorescence detector. Based on this measurement, the acquisition software was set up to trigger at 24 arbitrary units (a.u.) SSC, which is equivalent to an SSC cross section of 6 nm^2 (Rosetta Calibration, v1.23 Exometry, Amsterdam, The Netherlands).

1.9 Flow rate quantification

Each measurement day the flow rate was validated internally (Apogee calibration beads, Apogee Flow Systems, Hemel Hempstead, UK). Because the A60-Micro is equipped with a

syringe pump with volumetric control, we assumed a flow rate of 3.01 µL/min for all measurements.

1.10 Fluorescence calibration

Table S2 shows an overview of the beads used for fluorescence calibration. Calibration of the fluorescence detectors from arbitrary units (a.u.) to molecules of equivalent soluble fluorochrome (MESF) was accomplished using 2 µm QuantiBRITE-APC and PE beads (APC 2321-175 and 2364-87; PE 2364-89; both BD) and SPHERO Easy Calibration Fluorescent Particles (AK01, Spherotech Inc., Irma Lee Circle, IL, USA).

Calibration of the APC and PE detector was performed twice in the time period of these experiments. For each measurement, we added fluorescent intensities in MESF to the flow cytometry data files by custom-build software (MATLAB R2020b) using the following equation:

$$\mathrm{I(MESF)} = 10^{a \cdot \log_{10} \mathrm{I(a.u.)} + b} \qquad \text{Equation S1}$$

where I is the fluorescence intensity, and *a* and *b* are the slope and the intercept of the linear fits, respectively, see table S2.

At the second measurement day, the fluorescence calibration has been corrected for daily deviations using fluorescent beads.

1.11 Light scatter calibration

We used Rosetta Calibration to relate scatter measured by FSC or SSC to the scattering cross section and diameter of EVs. Fig. S1 shows print screens of the scatter calibrations. We modelled EVs as core-shell particles with a core refractive index of 1.38, a shell refractive index of 1.48, and a shell thickness of 6 nm. For each measurement, we added the FSC and SSC scattering cross sections and EV diameters to the flow cytometry datafiles by custom-build software (MATLAB R2020b).

1.12 MIFlowCyt checklist

The MIFlowCyt checklist is added to Table S3.

1.13 EV number concentration

The concentrations reported in the manuscript describe the number of particles (1) that exceeded the SSC threshold, corresponding to a side scattering cross section of 6 nm^2, (2) that were collected during time intervals, for which the count rate did not deviated more than 50% of the median count rate, (3) with a diameter <1,000 nm as measured by SSC after light scatter calibration (section 1.11) and (4) are positive for APC per mL of PDP.

Please note, the concentrations of EVs from activated platelets (CD62p-PE$^+$) have been left out of the manuscript, since for a part of the measurements the obtained number of counts was insufficient.

1.14 Data sharing

Data is available via: https://doi.org/10.6084/m9.figshare.21757067

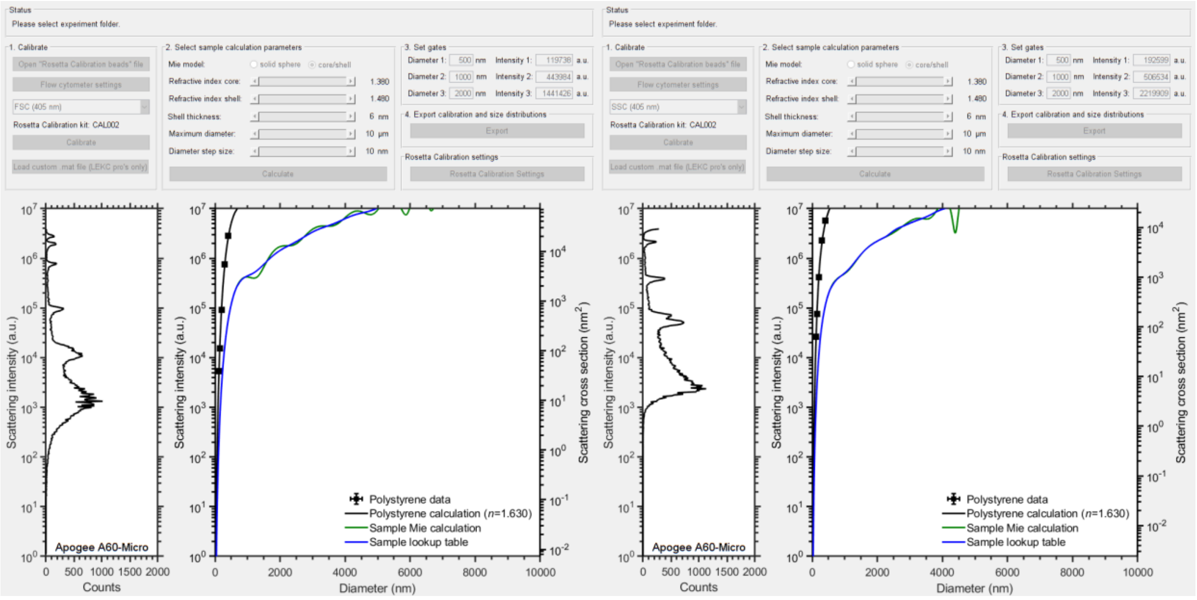

Figure S1. Scatter calibrations. (A) Forward scatter and (B) side scatter calibration of the A60-Micro by Rosetta Calibration. To relate scatter to the diameter of EVs, we modelled EVs as core-shell particles with a core refractive index of 1.38, a shell refractive index of 1.48, and a shell thickness of 6 nm.

Table S1: Overview of staining reagents. Characteristics being measured, analyte, analyte detector, reporter, isotype, clone, concentration, manufacturer, catalog number and lot number of used staining reagents. The antibody concentration during measurements was 11.25-fold lower than the antibody concentration during staining.

Characteristic measured	Analyte	Analyte detector	Reporter	Isotype	Clone	Concentration during staining (μg mL^{-1})	Manufacturer	Catalog number	Lot number
Integrin	Human CD61	Anti-human CD61 antibody	APC	IgG1	VI-PL2	8.3	Invitrogen-Thermo Fischer Scientific	17061942	2062626
Adhesion molecule	Human CD62p	Anti-human CD62P antibody	PE	IgG1	CLBThromb/6	0.89	Beckman Coulter	IM1759U	200051
Affinity for Fc receptor	Fc receptor	IgG1	APC	n.a.	MOPC-21	8.3	Beckman Dickinson	554681	9059624
	Fc receptor	IgG1	PE	n.a.	X40	0.89	Beckman Dickinson	345816	9035605

APC: allophycocyanin; CD: cluster of differentiation; IgG: immunoglobulin G; PE: phycoerythrin.

Table S2: Overview of fluorescence calibrations.

	Calibration date	Slope	Intercept	R^2
APC	2021-07-15	1.1573	-2.4199	0.9984
	2022-03-01	1.1936	-2.4899	0.9949
PE	2021-07-15	1.0118	-1.4839	0.9995
	2022-03-29	1.2538	-2.7574	0.9916
	2022-06-09*		-2.5630	Daily correction

APC: allophycocyanin; PE: phycoerythrin.

Table S3. MIFlowCyt checklist.

Requirement	Please Include Requested Information
1.1. Purpose	To investigate whether swarm detection in extracellular vesicle (EV) flow cytometry (FCM) can be prevented based on either a fixed dilution or a maximum count rate, dilution series of five different patient samples were measured. Both the total particle concentration as well as the concentration of platelet-derived EVs was of interest. We investigated under which circumstances these concentrations remained stable, and hence were not affected by swarm detection and defined a set of criteria based on that.
1.2. Keywords	Biomarkers, extracellular vesicles, flow cytometry, plasma
1.3. Experiment variables	Dilution factor
1.4. Organization name and address	Amsterdam University Medical Centers<br>Location Academic Medical Centre<br>Meibergdreef 9<br>1105 AZ Amsterdam<br>The Netherlands
1.5. Primary contact name and email address	Naomi Buntsma, n.c.buntsma@amsterdamumc.nl
1.6. Date or time period of experiment	August till October 2021<br>September 2022.
1.7. Conclusions	For our clinical samples and flow cytometer, swarm detection can be prevented when blood plasma samples are at least $1.1 \cdot 10^2$-fold diluted and measured at a count rate below $1.1 \cdot 10^4$ events s^{-1}.
1.8. Quality control measures	All samples were measured using an autosampler, which facilitates subsequent measurements of samples in a 96-well plate. Each well plate contained buffer-only controls (section S1.4), antibody in buffer controls (section S1.5), unstained controls (section S1.6) and isotype controls (section S1.7). The flow rate is validated daily. Fluorescence detectors were calibrated (section S1.10) with 2 µm Q-APC beads (2321-175 and 2364-87, BD), Q-PE beads (2364-89, BD) and SPHERO Easy

	Calibration Fluorescent Particles (AK01, Spherotech Inc., Irma Lee Circle, IL, USA). FSC and SSC were calibrated with Rosetta Calibration (v1.23, section S1.11).
1.9 Other relevant experiment information	Samples were measured on 4 different days, in a time period of 3 months in 2021 and a single measurement day in 2022.
2.1.1.1. Sample description	Thawed plasma samples from hospitalized humans after first acute myocardial infarction were used, which originated from the clinical study AFFECT-EV (NCT02931045). The five samples that were selected for this study, were collected at the first time point in the clinical study, which was at randomization.
2.1.1.2. Biological sample source description	Blood was collected in 7.5 mL EDTA tubes (S-Monovette, Sarstedt) via antecubital venipuncture using a 19-Gauge needle. Within 15 minutes from blood collection, plasma was prepared by double centrifugation at 2,500 g for 15 minutes at room temperature, acceleration speed 9, brake 1 (Hettich Zentrifugen, Tuttlingen, Germany). The first centrifugation spin was performed in blood collection spin, after which supernatant was collected up to 10 mm above the buffy coat and transferred to 15 mL polypropylene tubes (Greiner Bio-One B.V.). After the second spin, the supernatant was collected up to 5 mm above the pellet, transferred to 5 mL tubes and mixed by pipetting before transfer to 1.5 mL Eppendorfs in which they were freeze-stored at -80 ^{o}C. Before flow cytometry measurements, samples were thawed for 1 minute and 30 seconds at 37°C.
2.1.1.3. Biological sample source organism description	Samples are from patients and controls from the clinical research study AFFECT-EV (NCT02931045), which included hospitalized human patients after first acute myocardial infarction.
2.2. Sample characteristics	Plasma is expected to contain EVs, lipoproteins and proteins. Residual platelets might be present.
2.3. Sample treatment description	Please see section S1.3 for staining procedure.
2.4. Fluorescence reagent(s) description	Please see Table S1.
3.1. Instrument manufacturer	Apogee, Hemel Hempstead, UK

3.2. Instrument model	A60-Micro
3.3. Instrument configuration and settings	Samples were analyzed for 2 minutes at a flow rate of 3.01 μL·min^{-1} on an A60-Micro, equipped with a 405 nm laser (100 mW), 488 nm laser (150 mW) and 638 nm laser (150 mW). The trigger threshold was set at SSC 24 a.u., corresponding to an SSC cross section of 6 nm^2 (Rosetta Calibration).<br>For FSC and SSC, the PMT voltages were 470 V and 375 V, respectively. For all detectors, the peak height was analyzed. APC signals were collected with the 638-D Red(Peak) detector (long pass 650 nm filter, PMT voltage 480 V). PE signals were collected with the 488-Orange(Peak) detector (575/30 nm band pass filter, PMT voltage 450 V).
4.1. List-mode data files	Data is available via https://doi.org/10.6084/m9.figshare.21757067
4.2. Compensation description	No compensation was required because no fluorophore combinations were used that have overlapping emission spectra.
4.3. Data transformation details	Fluorescence detectors were calibrated (section S1.10) with 2 μm Q-APC beads (2321-175 and 2364-87, BD) , Q-PE beads (2364-89, BD) and SPHERO Easy Calibration Fluorescent Particles (AK01, Spherotech Inc., Irma Lee Circle, IL, USA).<br>FSC and SSC were calibrated with Rosetta Calibration (v1.23, section S1.11). The concentrations reported in the manuscript describe the number of particles that fulfil the gating criteria per mL.
4.4.1. Gate description	To automatically apply gates, generate pdf reports with scatter plots, and summarize the data in a table, custom-build software (MATLAB R2020b) was used. First, events that were collected during seconds for which the count rate deviated less than 50% from the median count rate were included. Second, events with a diameter <1,000 nm as measured by SSC after light scatter calibration (section S1.11) were included. Third, events positive for APC were included.
4.4.2. Gate statistics	The number of positive events was corrected for flow rate, measurement time and dilutions performed during sample preparation.

	Since the number of positive events for PE was on general quite low, we decided to exclude this from our analysis.
4.4.3. Gate boundaries	The lower boundaries of the fluorescent gates were automatically determined (MATLAB R2020b). For samples this was 61 MESF for CD61-APC and 96 MESF for PE -although the data of the latter was excluded from the analysis.

a.u.: arbitrary units; EDTA: ethylenediaminetetraacetic acid; EVs: extracellular vesicles; FSC: forward scattering; SSC: side scattering.
